# Withaferin A-Induced Apoptosis in Human Breast Cancer Cells Is Mediated by Reactive Oxygen Species

**DOI:** 10.1371/journal.pone.0023354

**Published:** 2011-08-10

**Authors:** Eun-Ryeong Hahm, Michelle B. Moura, Eric E. Kelley, Bennett Van Houten, Sruti Shiva, Shivendra V. Singh

**Affiliations:** 1 Department of Pharmacology & Chemical Biology, University of Pittsburgh School of Medicine, Pittsburgh, Pennsylvania, United States of America; 2 University of Pittsburgh Cancer Institute, Pittsburgh, Pennsylvania, United States of America; 3 Department of Anesthesiology, University of Pittsburgh School of Medicine, Pittsburgh, Pennsylvania, United States of America; 4 Vascular Biology Institute, University of Pittsburgh School of Medicine, Pittsburgh, Pennsylvania, United States of America; Roswell Park Cancer Institute, United States of America

## Abstract

Withaferin A (WA), a promising anticancer constituent of Ayurvedic medicinal plant *Withania somnifera*, inhibits growth of MDA-MB-231 and MCF-7 human breast cancer cells in culture and MDA-MB-231 xenografts *in vivo* in association with apoptosis induction, but the mechanism of cell death is not fully understood. We now demonstrate, for the first time, that WA-induced apoptosis is mediated by reactive oxygen species (ROS) production due to inhibition of mitochondrial respiration. WA treatment caused ROS production in MDA-MB-231 and MCF-7 cells, but not in a normal human mammary epithelial cell line (HMEC). The HMEC was also resistant to WA-induced apoptosis. WA-mediated ROS production as well as apoptotic histone-associated DNA fragment release into the cytosol was significantly attenuated by ectopic expression of Cu,Zn-superoxide dismutase in both MDA-MB-231 and MCF-7 cells. ROS production resulting from WA exposure was accompanied by inhibition of oxidative phosphorylation and inhibition of complex III activity. Mitochondrial DNA-deficient Rho-0 variants of MDA-MB-231 and MCF-7 cells were resistant to WA-induced ROS production, collapse of mitochondrial membrane potential, and apoptosis compared with respective wild-type cells. WA treatment resulted in activation of Bax and Bak in MDA-MB-231 and MCF-7 cells, and SV40 immortalized embryonic fibroblasts derived from Bax and Bak double knockout mouse were significantly more resistant to WA-induced apoptosis compared with fibroblasts derived from wild-type mouse. In conclusion, the present study provides novel insight into the molecular circuitry of WA-induced apoptosis involving ROS production and activation of Bax/Bak.

## Introduction

More than 40,000 women die from breast cancer each year in the United States alone despite significant advances towards targeted therapies and screening efforts [1]. Previous research has identified some of the risk factors associated with breast cancer, including family history, Li-Fraumeni syndrome, atypical hyperplasia of the breast, late-age at first full-term pregnancy, early menarche, and late menopause [2], [3]. Because some of these risk factors are not easily adjustable (*e.g.*, genetic predisposition), novel strategies for reduction of breast cancer risk are clinically desirable. This objective is partially fulfilled with selective estrogen-receptor (ER) modulators (*e.g.,* tamoxifen and raloxifene). Unfortunately, this approach is effective only against ER-positive breast cancers [4], [5]. In addition, selective ER modulators have adverse side effects including uterine cancer, thromboembolism, cataracts, and perimenopausal symptoms [4]. Therefore novel agents that can suppress growth of breast cancer cells regardless of ER status are clinically attractive. Natural products have received increased attention in recent years for the discovery of novel cancer chemopreventive and therapeutic agents [6].

*Withania somnifera* L. Dunal (commonly known as Ashwagandha or Indian winter cherry) has been used safely for thousands of years in Ayurvedic medicine practice for the treatment of various disorders. *Withania somnifera* exhibits a variety of pharmacological effects in experimental animals [7]–[11]. For example, administration of 50 mg/kg *Withania somnifera* extract for 1 month conferred cardioprotection against ischemia reperfusion injury in rats [8]. Markers of 6-hydroxydopamine-induced Parkinsonism were reversible in rats after gavage of *Withania somnifera* [9].

Anticancer effects for *Withania somnifera* and its constituents have also been described [12]–[26]. Anticancer effect of *Withania somnifera* is attributed to withanolides including withaferin A (WA). WA was shown to inhibit NF-κB-regulated gene expression in cancer cells [14]. Treatment with WA inhibited human umbilical vein endothelial cell sprouting at doses relevant to NF-κB inhibitory activity [16]. Recent studies, including those from our laboratory, have revealed proapoptotic effects of WA [19]–[21], [23], [24]. For example, WA was shown to trigger Par-4 dependent apoptosis in human prostate cancer cells [19]. Our own work has revealed that WA inhibits growth of MDA-MB-231 and MCF-7 human breast cancer cells by causing FOXO3a-Bim-dependent apoptosis [21]. We showed further that WA can trigger apoptosis and largely inhibit cell migration/invasion of breast cancer cells even after interleukin-6-induced activation of Signal Transducer and Activator of Transcription 3 [26], which should be viewed as a therapeutic advantage because this transcription factor is often hyperactive in human breast cancers.

Despite these advances, however, the molecular circuitry of WA-induced apoptosis is not fully defined. The present study fills this gap in our knowledge using MDA-MB-231 (an ER-negative cell line with mutant p53) and MCF-7 (an ER-positive cell line with wild-type p53) human breast cancer cells and their respective Rho-0 variants as models. We provide experimental evidence to implicate reactive oxygen species (ROS) in WA-induced apoptosis.

## Results

### WA treatment causes ROS production in human breast cancer cells

**Figure 1:**
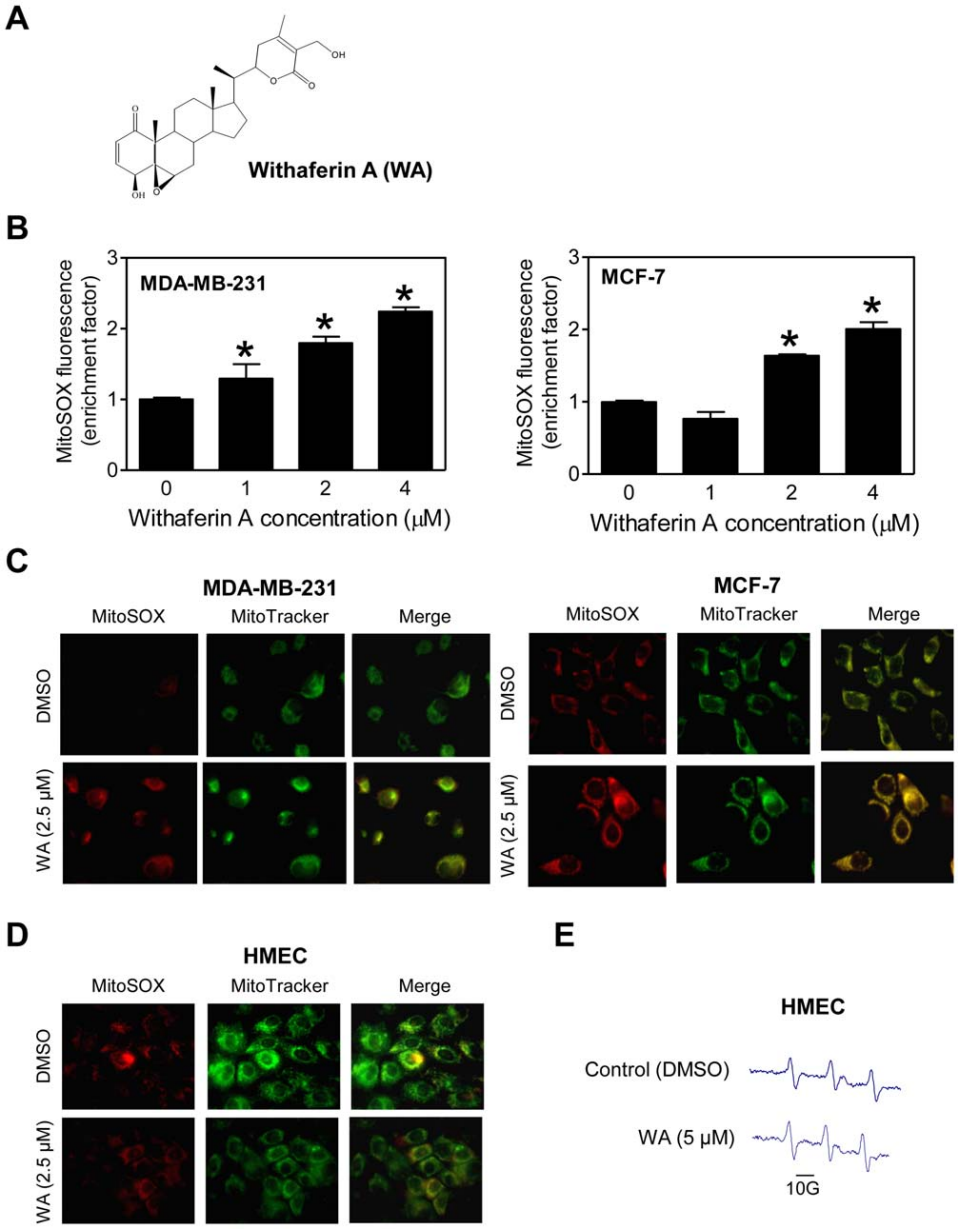
Withaferin A (WA) treatment causes reactive oxygen species (ROS) production in human breast cancer cells. (A) Chemical structure of WA. (B) Flow cytometric analysis for MitoSOX Red fluorescence in DMSO-treated control and WA-treated MDA-MB-231 and MCF-7 cells (4 h treatment). Results are shown as enrichment factor relative to DMSO-treated control (mean ± SD, *n* = 3). *P<0.05, significantly different compared with DMSO-treated control by one-way ANOVA with Dunnett's adjustment. (C) Fluorescence microscopy for MitoSOX Red fluorescence in MDA-MB-231 and MCF-7 cells following 4 h treatment with DMSO or 2.5 µM WA. (D) Fluorescence microscopy for MitoSOX Red fluorescence in HMEC following 4 h treatment with DMSO or 2.5 µM WA. (E) Representative EPR spectra in HMEC treated for 4 h with DMSO or 5 µM WA. All experiments were repeated at least twice.

Because ROS are implicated in apoptosis induction by a variety of natural anticancer agents [27], we questioned if proapoptotic response to WA (structure of WA is shown in Fig. 1A) was mediated by ROS generation. We tested this possibility using a chemical probe (MitoSOX Red) that accumulates in mitochondria and reacts with mitochondria-generated superoxide. As shown in Fig. 1B, WA treatment caused a dose-dependent increase in MitoSOX Red fluorescence in MDA-MB-231 and MCF-7 cells. In time-course kinetic experiments using 2.5 or 5 µM WA, increase in MitoSOX Red fluorescence over dimethyl sulfoxide (DMSO)-treated control was evident as early as 1–2 h after treatment and persisted for at least 4–6 h (results not shown). ROS generation by WA treatment in MDA-MB-231 and MCF-7 cells was confirmed by fluorescence microscopy after staining the cells with MitoSOX Red. As shown in Fig. 1C, DMSO-treated control MDA-MB-231 and MCF-7 cells exhibited weak and diffuse MitoSOX Red fluorescence. On the other hand, cells treated for 4 h with 2.5 µM WA were brightly stained with MitoSOX Red suggesting superoxide generation. Moreover, MitoSOX Red-associated fluorescence co-localized with the MitoTracker Green signal indicating mitochondrial origin of WA-induced superoxide generation (Fig. 1C). We next designed experiments to determine susceptibility of a normal human mammary epithelial cell line (HMEC) to ROS production by WA. Interestingly, HMEC appeared resistant to ROS production by WA as judged by fluorescence microscopy using MitoSOX Red (Fig. 1D) and electron paramagnetic resonance (EPR) using a cell-permeable spin probe 1-hydroxy-3-methoxy-carbonyl-2,2,5,5-tetramethylpyrrolidine (Fig. 1E). These results indicated that while WA treatment caused ROS production in breast cancer cells, HMEC were resistant to pro-oxidant effect of this agent. These results are noteworthy because selectivity towards cancer cells is a highly desirable feature for potential cancer chemopreventive agents.

### Cu,Zn-Superoxide dismutase (Cu,Zn-SOD) overexpression confers protection against WA-induced ROS production and apoptosis

**Figure 2:**
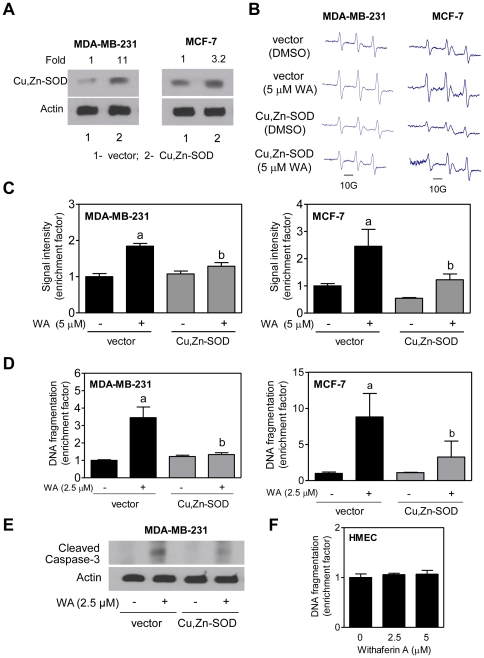
Cu,Zn-Superoxide dismutase (Cu,Zn-SOD) overexpression attenuates withaferin A (WA)-induced apoptosis in MDA-MB-231 and MCF-7 cells. (A) Immunoblotting for Cu,Zn-SOD using lysates from MDA-MB-231 or MCF-7 cells stably transfected with empty vector (lane 1) or vector encoding for Cu,Zn-SOD (lane 2). (B) Representative EPR spectra in MDA-MB-231 and MCF-7 cells stably transfected with empty vector or vector encoding for Cu,Zn-SOD and treated for 4 h with DMSO or 5 µM WA. (C) Quantitation of EPR signal intensity in MDA-MB-231 and MCF-7 cells transfected with empty vector or vector encoding for Cu,Zn-SOD and treated for 4 h with DMSO or 5 µM WA. Results shown are mean ± SD (*n* = 3). (D) Histone-associated DNA fragment release into the cytosol (a measure of apoptosis) in MDA-MB-231 and MCF-7 cells transfected with empty vector or vector encoding for Cu,Zn-SOD and treated for 24 h with DMSO or WA. Results shown are mean ± SD (*n* = 3). (E) Immunoblotting for cleaved caspase-3 using lysates from MDA-MB-231 cells stably transfected with empty vector or vector encoding for Cu,Zn-SOD and treated for 24 h with DMSO or WA. (F) Histone-associated DNA fragment release into the cytosol in HMEC treated for 24 h with DMSO or WA. Results shown are mean ± SD (*n* = 3). Significantly different (*P*<0.05) compared with ^a^control (DMSO-treated), and ^b^ between groups at each dose by one-way ANOVA followed by Bonferroni's adjustment. For data in panels C,D, and F, data are shown as enrichment factor relative to DMSO-treated control. All experiments were repeated at least twice.

We designed experiments involving stable overexpression of Cu,Zn-SOD to confirm the role of ROS in proapoptotic response to WA. Protein level of Cu,Zn-SOD was about 11- and 3.2-fold higher in MDA-MB-231 and MCF-7 cells, respectively, that were stably transfected with the Cu,Zn-SOD expression plasmid in comparison with empty vector-transfected control cells (Fig. 2A). Fig. 2B depicts typical EPR spectra in MDA-MB-231 and MCF-7 cells transfected with empty vector or vector encoding for Cu,Zn-SOD after 4 h treatment with DMSO (control) or 5 µM WA. Radical signal intensity was very weak in DMSO-treated cells. However, WA-mediated increase in EPR signal intensity was suppressed significantly by stable overexpression of Cu,Zn-SOD in both cell lines (Fig. 2C). Empty vector-transfected cells exhibited significant enrichment of histone-associated apoptotic DNA fragment release into the cytosol upon treatment with WA when compared with corresponding DMSO-treated controls (Fig. 2D). WA-mediated apoptotic DNA fragmentation (Fig. 2D) and cleavage of procaspase-3 (Fig. 2E) was markedly attenuated in cells with stable overexpression of Cu,Zn-SOD (caspase-3 is not expressed in MCF-7 cells). Consistent with EPR data (Fig. 1E), HMEC were completely resistant to WA-induced histone-associated apoptotic DNA fragment release into the cytosol (Fig. 2F). Two critical conclusions are discernible from these experiments: (a) ROS play a critical role in WA-induced apoptosis, and (b) HMEC are resistant to apoptosis induction by WA treatment.

### WA treatment inhibits oxidative phosphorylation in human breast cancer cells

**Figure 3:**
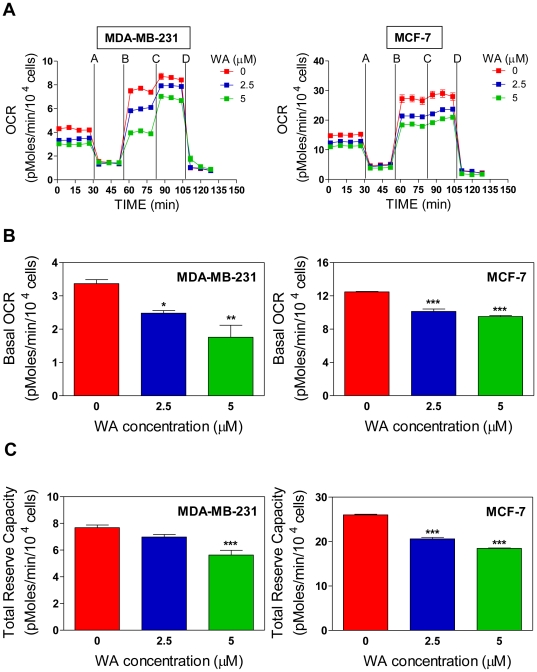
Withaferin A (WA) treatment inhibits oxidative phosphorylation (OXPHOS) in MDA-MB-231 and MCF-7 cells. (A) Pharmacologic profiling of oxygen consumption rate (OCR), an indicator of OXPHOS, in MDA-MB-231 and MCF-7 cells following 4 h treatment with DMSO (control) or the indicated concentrations of WA. After measurement of basal OCR, the cells were treated with a series of metabolic inhibitors, including oligomycin (injection A); FCCP (injection B); 2-DG (injection C); and rotenone (injection D) at the indicated times. (B) Effect of WA treatment on basal OCR in MDA-MB-231 and MCF-7 cells. Basal OCR was calculated using the difference between the mean of time points prior to injection A (#1–#4) and after injection D (#14 to #16; rotenone-sensitive) (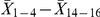). (C) Effect of WA treatment on total reserve respiration capacity in MDA-MB-231 and MCF-7 cells. Total reserve respiration capacity was calculated using the mean of the time points after injection C (#11–#13) minus the mean of time points after injection D (#14–#16) (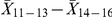). Data shown are mean ± SEM of three independent experiments, each performed in triplicate. *P<0.05; **P<0.01; and ***P<0.001, significantly different compared with control by one-way ANOVA with Dunnett's adjustment.

Because mitochondria are the major source of cellular superoxide, which result from incomplete reduction of oxygen from 0.1–0.2% of the “leaky” electrons that escape normal oxidative phosphorylation, we next tested the possibility whether WA affected mitochondrial bioenergetics. We measured oxygen consumption rate (OCR), an indicator of oxidative phosphorylation (OXPHOS), in MDA-MB-231 and MCF-7 cells (Fig. 3A). Basal OCR was markedly higher in the MCF-7 cells in comparison with MDA-MB-231 (Fig. 3B). Both cells showed a significant decrease in basal OCR after a 4 h treatment with WA (Fig. 3B). However, WA-mediated decrease in basal OXPHOS was more pronounced in the MDA-MB-231 cell line compared with MCF-7. The spare and total reserve capacity for OXPHOS in these two cell lines were examined by sequential exposure to four metabolic inhibitors: oligomycin (identified by A in Fig. 3A), carbonyl cyanide 4-trifluoromethoxyphenylhydrazone (FCCP) (identified by B in Fig. 3A), 2-deoxyglucose (2-DG) (identified by C in Fig. 3A), and rotenone (identified by D in Fig. 3A) [28]. Addition of oligomycin, which is an inhibitor of F_1_F_0_-ATPase complex V, caused an inhibition of electron flow. As a consequence, oligomycin addition decreased the OCR (Fig. 3A), which is an indicator of oxygen linked ATP production. FCCP treatment allows maximum oxygen consumption and gives a measure of spare respiratory capacity. After injection of 2-DG, a glucose analog that inhibits hexokinase and glucose utilization by glycolysis, cells showed an increase in OCR as a result of activation of the total reserve respiratory capacity. Finally, addition of the complex I inhibitor rotenone arrested electron flow through the mitochondrial respiratory complexes and caused a dramatic decrease in the oxygen consumption, as evidenced by the drop in the OCR in both cell lines (Fig. 3A). Total reserve capacity for OXPHOS was measured by the mean of points after 2-DG injection minus the mean of points after rotenone injection. WA treatment resulted in a statistically significant decrease in reserve OXPHOS in MDA-MB-231 and MCF-7 cells especially at the 5 µM concentration (Fig. 3C).

**Figure 4:**
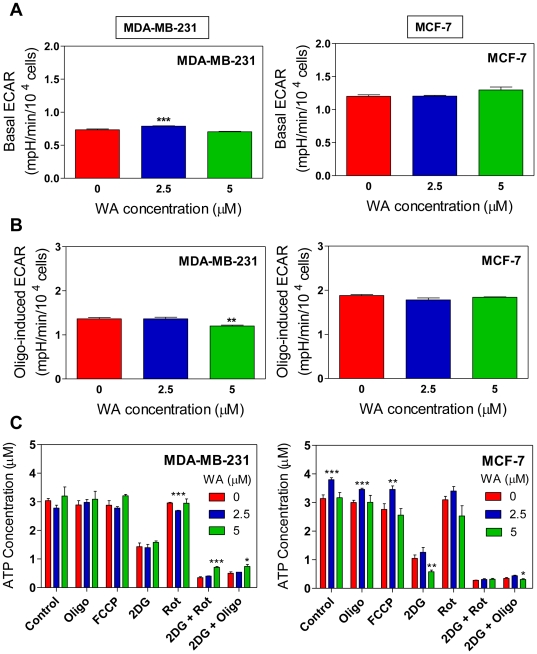
Withaferin A (WA) treatment fails to alter acidification rate (lactate production)and steady-state levels of ATP. (A) Effect of WA treatment on basal extracellular acidification rate (ECAR), a measure of lactate production, in MDA-MB-231 and MCF-7 cells following 4 h treatment with DMSO (control) or the indicated concentrations of WA. (B) Effect of WA treatment on oligomycin-induced (oligo-induced) ECAR in MDA-MB-231 and MCF-7 cells. Results shown are mean ± SEM of three independent experiments, each performed in triplicate. (C) Steady-state levels of ATP in MDA-MB-231 and MCF-7 cells treated with DMSO (control) or the indicated concentrations of WA in the absence or presence of the metabolic inhibitors. Results shown are mean ± SEM of combination of three independent experiments, each performed in quadruplicate. *P<0.05; **P<0.01; and ***P<0.001, significantly different compared with control by one-way ANOVA with Dunnett's adjustment (Panels A and B) or Student's *t-*test (panel C).

Fig. 4A depicts basal extracellular acidification rate (ECAR), an indicator of lactate production, for MDA-MB-231 and MCF-7 cells after treatment with DMSO (control) or WA (2.5 or 5 µM). Basal lactate production in MDA-MB-231 and MCF-7 cells was either not altered or slightly but significantly increased (MDA-MB-231 cells at 2.5 µM WA dose) by WA treatment compared with control. Oligomycin-induced ECAR (Fig. 4B) was only modestly inhibited by 5 µM WA treatment in MDA-MB-231 cells only (Fig. 4B). However, it is difficult to predict whether such a small difference, albeit statistically significant, is biologically meaningful.

Because WA treatment caused a decrease in OXPHOS without a compensating increase in glycolysis, a decrease in steady-state levels of ATP was anticipated. However, steady-state levels of ATP were unaffected by WA treatment in either cell line (Fig. 4C). These observations suggest that WA treatment possibly causes a decline in ATP utilization. Taken together, these results indicated that OXPHOS was significantly inhibited by WA treatments in both cell lines.

### WA inhibits complex III activity in MDA-MB-231 cells

**Figure 5:**
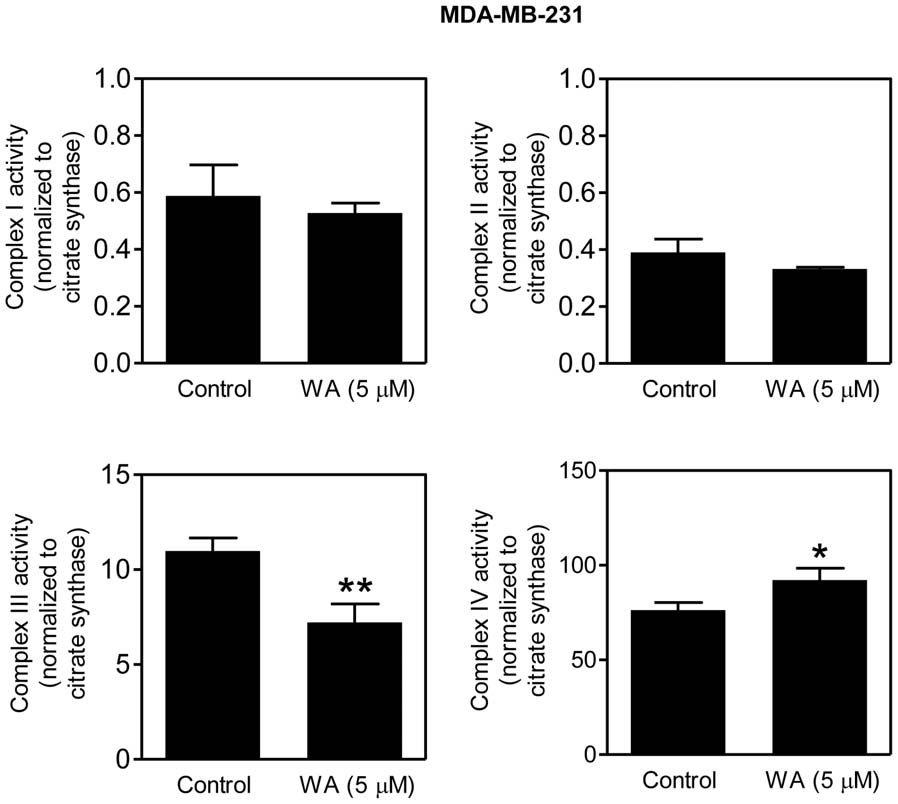
WA treatment inhibits complex III activity in MDA-MB-231 breast cancer cells. Mitochondrial complex enzyme activities were determined using lysates from MDA-MB-231 cells treated for 6 h with DMSO or 5 µM WA. Results shown are mean ± SD (*n* = 3). *P<0.05; **P<0.01, significantly different compared with control by Student's *t*-test.

Because WA treatment exerted a relatively greater effect on OXPHOS in the MDA-MB-231 cells, we used this cell line to determine whether WA-mediated inhibition of OXPHOS was accompanied by inhibition of mitochondrial respiratory chain complex activities. Treatment of MDA-MB-231 cells with 5 µM WA for 6 h resulted in a statistically significant decrease in the activity of complex III, but not of complex I or complex II (Fig. 5). A modest but significant increase in the activity of complex IV was also observed in the WA-treated MDA-MB-231 cells relative to DMSO-treated control (Fig. 5).

### Rho-0 variants of MDA-MB-231 and MCF-7 cells are resistant to WA-induced apoptosis

**Figure 6:**
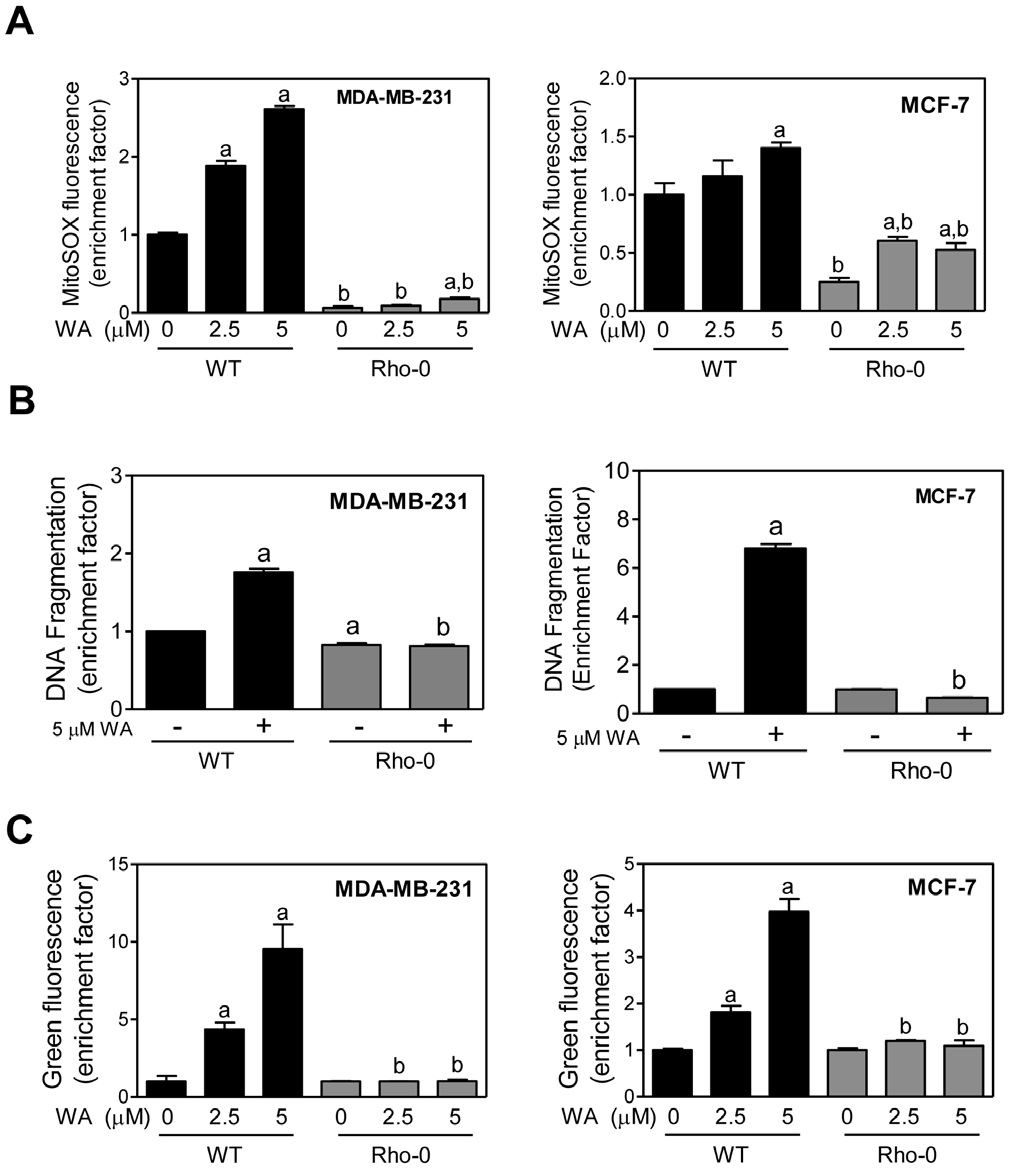
Rho-0 variants of MDA-MB-231 and MCF-7 cells are resistant to withaferin A (WA)-mediated apoptosis. (A) MitoSOX Red fluorescence (a measure of ROS production) in wild-type (WT) and Rho-0 variants of MDA-MB-231 and MCF-7 cells following 4 h treatment with DMSO (control) or WA. Enrichment of MitoSOX Red fluorescence relative to DMSO-treated wild-type cells is shown for both MDA-MB-231 and MCF-7 cells. (B) Histone-associated DNA fragment release into the cytosol in wild-type and Rho-0 variants of MDA-MB-231 and MCF-7 cells following 24 h treatment with DMSO or WA. Enrichment relative to DMSO-treated wild-type cells is shown for both MDA-MB-231 and MCF-7 cells. (C) Analysis of mitochondrial membrane potential (monomeric JC-1-associated green fluorescence) in wild-type and Rho-0 variants of MDA-MB-231 and MCF-7 cells following 24 h treatment with DMSO or WA. Enrichment relative to DMSO-treated wild-type cells is shown for both MDA-MB-231 and MCF-7 cells. Results shown are mean ± SD (*n* = 3). Significantly different (*P*<0.05) compared with corresponding ^a^control (DMSO-treated) and ^b^between groups at each dose by one-way ANOVA followed by Bonferroni's multiple comparison test. Each experiment was repeated at least twice.

To get further support for the contribution of mitochondrial ROS in WA-mediated apoptosis, we generated Rho-0 variants of MDA-MB-231 and MCF-7 cells as described by us previously [29]. Of the 13 polypeptides encoded by the mitochondrial DNA, many are integral components of the mitochondrial respiratory chain complexes [30]. The Rho-0 cells lack OXPHOS but their survival is dependent on ATP derived from anaerobic glycolysis. The Rho-0 variants of MDA-MB-231 and MCF-7 cells have significantly diminished levels of complex I and III activity [29]. As expected, exposure of wild-type MDA-MB-231 and MCF-7 cells to WA for 4 h resulted in a statistically significant enrichment of MitoSOX Red fluorescence over DMSO-treated control (Fig. 6A). ROS production was greatly diminished in the Rho-0 cells by a similar treatment with WA (Fig. 6A). WA treatment (5 µM, 24 h) caused a significant increase in apoptotic DNA fragmentation in wild-type MDA-MB-231 and MCF-7 cells but not in the Rho-0 variants (Fig. 6B).

We used JC-1 to determine the effect of WA treatment on mitochondrial membrane potential. Cationic JC-1 enters mitochondria due to negative charge established by the intact mitochondrial membrane potential. Mitochondria of healthy cells are characterized by red fluorescence due to accumulation of J-aggregates. In apoptotic cells, JC-1 dye accumulates in the cytoplasm in monomeric form (green fluorescence) due to collapse of the mitochondrial membrane potential. WA treatment caused a dose-dependent accumulation of monomeric JC-1 in wild-type MDA-MB-231 and MCF-7 cells, but not in their respective Rho-0 variants (Fig. 6C). These results indicated that ROS acted upstream of mitochondrial membrane potential collapse in WA-induced apoptosis.

### WA treatment causes activation of Bak and Bax

**Figure 7:**
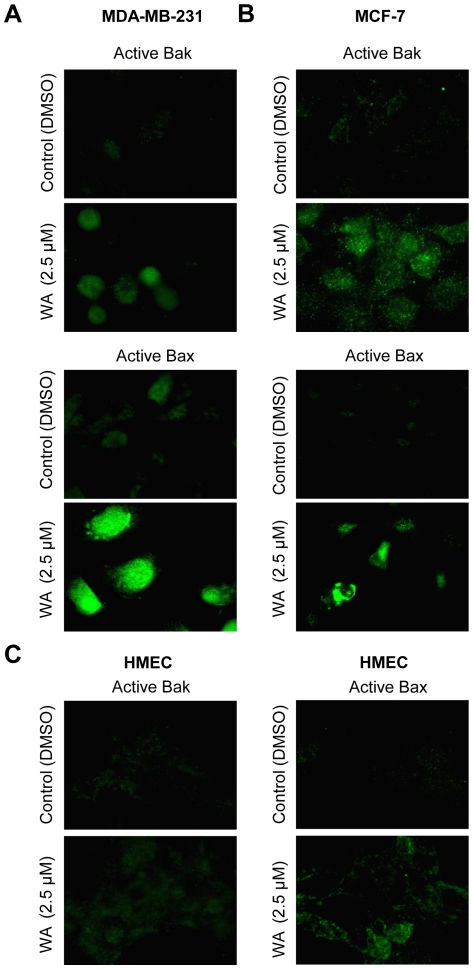
Withaferin A (WA) treatment causes activation of Bak and Bax in breast cancer cells. Immunofluorescence microscopy for active Bak and Bax in MDA-MB-231 (A), MCF-7 (B), and HMEC (C) following 24 h treatment with DMSO (control) or 2.5 µM WA. Each experiment was repeated at least twice.

**Figure 8:**
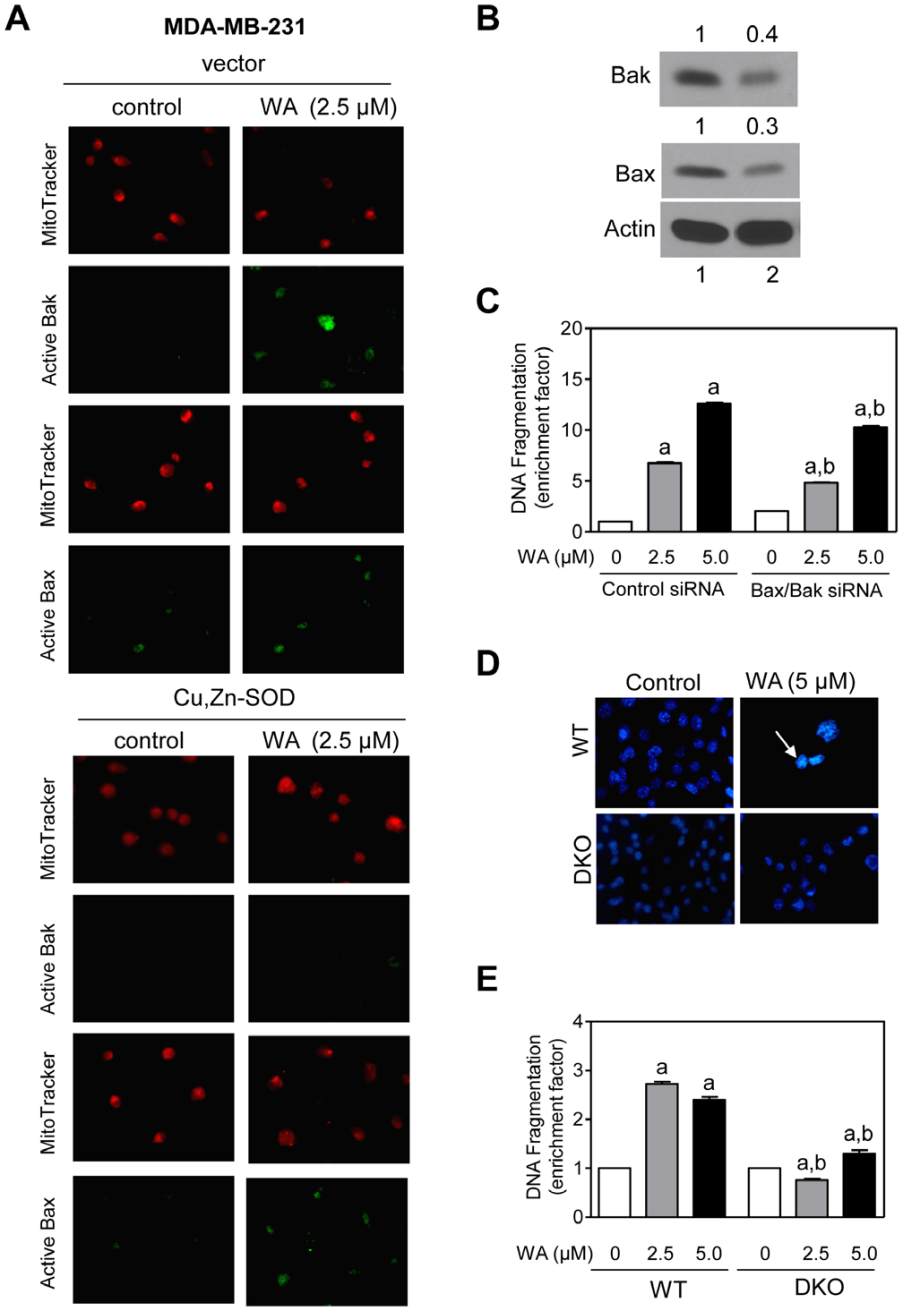
Bak and Bax are required for withaferin A (WA)-induced apoptosis. (A) Immunofluorescence microscopy for active Bak and Bax in MDA-MB-231 cells stably transfected with empty vector or vector encoding for Cu,Zn-SOD and treated for 24 h with DMSO or WA. (B) Immunoblotting for Bak and Bax using lysates from MCF-7 cells transiently transfected with a control nonspecific small interfering RNA (siRNA; lane 1) or Bax- or Bak-targeted siRNA (lane 2). (C) Histone-associated DNA fragment release into the cytosol in siRNA-transfected MCF-7 cells following 24 h treatment with DMSO (control) or the indicated concentrations of WA. Results are shown as enrichment factor relative to DMSO-treated control siRNA transfected cells (mean ± SD, *n* = 3). (D) Fluorescence microscopic analysis for apoptotic cells with condensed and fragmented DNA (DAPI assay) in SV40 immortalized mouse embryonic fibroblasts (MEF) derived from wild-type (WT) and Bax and Bak double knockout (DKO) mice and treated for 24 h with DMSO (control) or 5 µM WA. (E) Histone-associated DNA fragment release into the cytosol in WT and DKO treated for 24 h with DMSO (control) or the indicated concentrations of WA. Results are shown as enrichment factor relative to DMSO-treated wild-type MEF (mean ± SD, *n* = 3). Significantly different (*P*<0.05) compared with ^a^DMSO-treated control siRNA-transfected MCF-7 cells (panel C) or DMSO-treated WT MEF (panel E), and ^b^between groups at each dose by one-way ANOVA followed by Bonferroni's test. Similar results were observed in two independent experiments.

Results presented thus far establish a critical role for ROS in proapoptotic effect of WA, but do not shed light on mechanisms downstream of ROS production in the execution of WA-induced apoptosis. Because oxidative stress was previously shown to cause activation (conformational change) of Bax [29], [31], we tested the possibility of ROS-dependent Bax activation in our model. We tested this possibility by examining the effect of WA treatment on activation of Bak and Bax by immunofluorescence microscopy using antibodies that recognize only activate forms of these proteins. DMSO-treated control MDA-MB-231 (Fig. 7A) and MCF-7 cells (Fig. 7B) exhibited very weak activate Bak- or Bax-associated immunofluorescence. Treatment of MDA-MB-231 and MCF-7 cells with 2.5 µM (Fig. 7A-B) and 5 µM WA (results not shown) for 24 h resulted in enrichment of the activate Bak and Bax proteins. These results indicated that WA treatment caused activation of both Bax and Bak in MDA-MB-231 and MCF-7 cells. On the other hand, HMEC were resistant to Bak activation by WA, although a slight increase in active Bax-associated green fluorescence over DMSO-treated control was visible in WA-treated HMEC (Fig. 7C). Furthermore, when ROS production in the MDA-MB-231 cells was suppressed by overexpression of Cu,Zn-SOD, level of active Bak, but not Bax, was markedly suppressed (Fig. 8A). These results indicated that while WA treatment caused activation of both Bax and Bak, only Bak activation was dependent on ROS production.

### Bak and Bax are essential for apoptotic response to WA

We utilized siRNA to further probe into the role of Bax and Bak in regulation of WA-induced apoptosis. Level of Bak and Bax protein was decreased by 60%–70% in MCF-7 cells transiently transfected with Bak and Bax-targeted siRNAs (Fig. 8B). WA treatment (24 h) caused a dose-dependent and statistically significant increase in histone-associated DNA fragment release into the cytosol in cells transfected with the control nonspecific siRNA. On the other hand, combined knockdown of Bax and Bak proteins conferred partial but significant protection against WA-induced DNA fragmentation (Fig. 8C). We reasoned that partial protection against WA-induced DNA fragmentation by combined RNA interference of Bax and Bak could be attributable to incomplete knockdown of these proteins. To address this question, we used SV40 immortalized mouse embryonic fibroblasts (MEF) derived from wild-type (WT) and Bax and Bak double knockout mice (DKO). As can be seen in Fig. 8D, WA treatment resulted in appearance of apoptotic cells with condensed and fragmented DNA in WT cells but not in DKO. Consistent with these observations, DKO cells were nearly completely resistant to WA-induced apoptotic DNA fragmentation (Fig. 8E). These results indicated that WA-induced apoptosis was regulated by Bax and Bak.

## Discussion

The primary objective of the present study was to gain insight into the mechanism by which WA, a highly promising herbal medicine constituent [32], triggers apoptosis in human breast cancer cells. We show that WA-induced apoptosis in human breast cancer cells is mediated by mitochondria-derived ROS as evidenced by flow cytometric and microscopic measurements using a chemical probe and EPR using a spin probe. ROS production by WA treatment is apparently not influenced by the p53 or ER status because pro-oxidant effect of WA is evident in both MDA-MB-231 and MCF-7 cells. Because stable overexpression of Cu,Zn-SOD significantly blunts apoptotic cell death response to WA, it is reasonable to conclude that the signal for initiation of WA-induced cell death is most likely provided by the ROS. Even though ROS generation by WA treatment was documented previously in HL-60 leukemia cells [20], the present study is the first to demonstrate that WA-induced ROS production is accompanied by dysfunction of mitochondria reflected by inhibition of both basal and reserve OXPHOS and inhibition of complex III activity. Resistance of Rho-0 cells to WA-mediated ROS generation as well as apoptosis provides further support to our contention that proapoptotic response to this agent is initiated by the mitochondria-derived ROS. Translational implication of these findings is that the anti-cancer response to WA may be compromised in the presence of antioxidants. However, the exact topology of mitochondrial superoxide production in WA-treated cells remains to be elucidated. Inhibition of OXPHOS at complex III is often associated with superoxide production within the mitochondrial matrix or the intermembrane space [33]. Superoxide generated in the intermembrane space would be susceptible to Cu,Zn-SOD which is found in this compartment.

It is interesting to note that despite significant inhibition of OXPHOS in both MDA-MB-231 and MCF-7 cells, steady-state levels of ATP are not altered by WA treatment in either cell line. Because rate of lactate production is comparable in control and WA-treated cells, it is reasonable to argue that maintenance of the ATP levels is not due to a compensatory increase in the glycolysis. Steady-state levels of ATP are regulated not only by its synthesis but also utilization by a variety of reactions including synthesis of macromolecules, and sodium and calcium cycling across the plasma membrane [34], [35]. Therefore, the possibility that WA treatment causes a decrease in bioenergetic demand for ATP cannot be ruled out. Further studies are needed to systematically explore this possibility.

Another critical objective of the present study was to gain insight into the mechanism downstream of ROS generation in execution of WA-mediated cell death. The Bcl-2 family proteins have emerged as critical regulators of the cell death by functioning as either inhibitors (*e.g.*, Bcl-2 and Bcl-xL) or facilitators (*e.g.*, Bax and Bak) of apoptosis [36], [37]. We have shown previously that WA treatment causes a modest increase in the levels of Bax and/or Bak proteins in MDA-MB-231 and MCF-7 cells [21]. WA-mediated induction of Bax in acute T leukemia cell line (MOLT-4) has also been reported [24]. To the contrary, induction of Bax upon treatment with WA was not evident in U937 human leukemia cells [23]. While involvement of Bax in WA-induced apoptosis seems cell line-specific, none of these studies examined the effect of WA treatment on activation of Bax and Bak. This is important because activation (conformational change) and mitochondrial translocation of Bax is required for its proapoptotic activity in response to different treatments including hyperoxia [31], [37], [38]. The present study indicates that WA treatment causes activation of both Bak and Bax. Moreover, both Bax and Bak seem to contribute to apoptosis induction by WA as evidenced by the results using MEF.

In conclusion, the present study demonstrates that WA treatment inhibits basal and reserve OXPHOS, but not glycolysis, leading to ROS production and apoptotic DNA fragmentation. Translational implication of these findings is that the anticancer effect of WA may be compromised in the presence of anti-oxidants. This is a strong possibility because cell death response to WA is significantly attenuated by overexpression of Cu,Zn-SOD.

## Methods

### Reagents

WA (purity ∼96%) was purchased from Chromadex (Irvine, CA), dissolved in DMSO, and diluted with complete media immediately before use. The 4′,6-diamidino-2-phenylindole (DAPI), FCCP, oligomycin, 2-DG, and rotenone were purchased from Sigma-Aldrich (St. Louis, MO). MitoSOX Red, MitoTracker Green, and MitoTracker Red were purchased from Invitrogen-Life Technologies (Carlsbad, CA). Cell-permeable spin probe 1-hydroxy-3-methoxy-carbonyl-2,2,5,5-tetramethylpyrrolidine was purchased from Noxygen Science Transfer and Diagnostics (Elzach, Germany). Anti-actin antibody was from Sigma-Aldrich; anti-activated Bak and anti-Cu,Zn-SOD antibodies were from EMD Chemicals (Gibbstown, NJ); and anti-activated Bax (6A7) antibody was from BD Biosciences (San Diego, CA).

### Cell lines

The MDA-MB-231 and MCF-7 cells were purchased from the American Type Culture Collection (Manassas, VA) and maintained as described by us previously [29], [39]. The Rho-0 variants of MDA-MB-231 and MCF-7 cells were generated and maintained as described by us previously [29]. The MDA-MB-231 or MCF-7 cells were transfected with empty pcDNA3.1 vector or pcDNA3.1 vector encoding for Cu,Zn-SOD using FuGENE6. Cells stably overexpressing Cu,Zn-SOD were selected by culture in medium supplemented with 800 µg/mL of G418 over a period of 8 weeks. The HMEC were maintained in serum-free Mammary Epithelial Growth Medium (Clonetics, San Diego, CA). The SV40 immortalized MEF from WT and DKO mice were maintained as described by us previously [40].

### Detection of ROS

ROS production was measured by flow cytometry or immunofluorescence microscopy following staining with MitoSOX Red and EPR. For flow cytometric analysis, cells were treated with DMSO (control) or desired concentrations of WA for specified time intervals and then incubated with 5 µM MitoSOX Red for 30 min. Cells were collected, washed with phosphate-buffered saline (PBS) and fluorescence was detected using a Coulter Epics XL Flow Cytometer. For immunofluorescence microscopy, cells were plated on coverslips and allowed to attach by overnight incubation. Cells were then treated with DMSO (control) or WA followed by incubation with 2.5 µM MitoSOX Red for 30 min at 37°C. Cells were then treated for 15 min with 200 nM MitoTracker Green to stain mitochondria. After washing with PBS, cells were fixed with 2% paraformaldehyde for 1 h at room temperature and examined under a Leica fluorescence microscope at 100× objective magnification. For EPR studies, one million cells were plated in 10-cm dish and exposed to DMSO or 5 µM WA. Cells were collected by scraping, centrifuged, and the pellet was re-suspended in 100 µL of Krebs HEPES buffer (pH 7.4). EPR was performed using a Bruker eScan Table Top EPR spectrometer. Briefly, samples were exposed to spin probe at a final concentration of 200 µM and immediately loaded into 50 µL glass capillaries and placed into a temperature and gas-controlled EPR cavity for 10 min. Control samples, in the absence of cell sample, were conducted to determine the auto-oxidation of the spin probe under our experimental conditions and were subtracted from the signal intensity values. The EPR instrument settings were as follows: field sweep 50G; microwave frequency 9.78 GHz; microwave power 20 mW; modulation amplitude 2G; conversion time 327 ms; time constant 655 ms; and receiver gain 1×10^5^. To minimize the deleterious effects of adventitious metals, all buffers were treated with Chelex resin.

### Immunoblotting

Preparation of total lysates from control or WA-treated cells was done as described by us previously [21], [26]. Western blotting was done as described by us previously [21], [26]. Immunoreactive bands were visualized using enhanced Chemiluminescence methods.

### Detection of apoptosis

Apoptosis induction was assessed by quantitation of histone-associated DNA fragment release into the cytosol using an ELISA kit (Roche Applied Science, Indianapolis, IN) or DAPI assay. For DAPI assay, cells were plated on glass coverslips in 12-well plates. After 24 h incubation, cells were treated with DMSO or 5 µM WA for an additional 24 h and fixed in 2% paraformaldehyde for 1 h at room temperature. Cells were permeabilized with 0.5% Triton X-100 for 10 min, and stained with 10 ng/mL DAPI for 10 min at room temperature. Apoptotic cells were counted under a Leica DC 300F fluorescence microscope.

### Real time measurements of OXPHOS and glycolysis

The OCR and ECAR, which respectively are the indicators of OXPHOS and glycolysis, were measured in real-time using a Seahorse Bioscience XF24 Extracellular Flux Analyzer (Billerica, MA) [41]. Cells were seeded in XF24-well microplate (4×10^4^ cells/well), and incubated at 37°C for 20–24 h. Cells were washed with unbuffered Dulbecco's modified essential medium (DMEM), and incubated for 1 h at 37°C without CO_2_ in 750 µL of unbuffered DMEM (pH 7.4) supplemented with GlutaMax-1 (final concentration 2 mM), sodium pyruvate (final concentration 1 mM), glucose (final concentration 25 mM), sodium chloride (final concentration 32 mM) and phenol red. After determining the basal rates of OCR and ECAR, a pharmacological profiling of mitochondrial function was performed by injection of four compounds that affect bioenergetics, including: oligomycin (final concentration 1 µM) at injection A, FCCP (final concentration 300 nM) at injection B, 2-DG (final concentration 100 mM) at injection C, and rotenone (final concentration 1 µM) at injection D. After completion of the experiment, cells were trypsinized and counted for normalization. The OCR was analyzed using an algorithm described by Gerencser *et al.* [42]. ATP measurements were done using the ATPlite kit from Perkin Elmer (Waltham, MA). This method is based on the amount of light produced by an ATP-dependent luciferase reaction. Briefly cells (4×10^4^ cells/well) were grown overnight in 96-well black microplate and treated separately in quadruplicate with the indicated test compounds for 45 min. The cells were lysed by adding 50 µL of mammalian cell lysis solution followed by addition of 50 µL of substrate solution. Light produced was measured using a Biotek Synergy 2 Plate Reader (Winooski, VT) and compared to a series of ATP standards.

### Measurements of mitochondrial complex activities and membrane potential

Control and WA-treated MDA-MB-231 cell pellets were frozen and thawed three times to lyse plasma membrane. Activities were detected spectrophotometrically as previously described [43]. Complex I activity was detected by the rotenone sensitive oxidation of NADH. Complex II and III activities were detected by the oxidation/reduction of ubiquinol. Complex IV activity was detected by the oxidation of cytochrome *c*. All activities were normalized to the activity of the matrix enzyme citrate synthase. The effect of WA treatment on mitochondrial membrane potential was determined using JC-1 assay kit from Invitrogen-Life Technologies as instructed by the supplier. The wild-type and Rho-0 variants of MDA-MB-231 and MCF-7 cells were allowed to attach by overnight incubation, exposed to WA for specified time, and collected by trypsinization. The cells were incubated with medium containing JC-1 (2 µM) for 15 min at 37°C. The cells were washed and re-suspended in PBS, and fluorescence was measured using a Coulter Epics XL Flow Cytometer.

### Immunofluorescence microscopy for detection of activate Bax and Bak

Desired cells treated with DMSO or WA (24 h) were exposed to 200 nM MitoTracker Red for 30 min, and fixed with paraformaldehyde following permeabilization with Triton X-100. Next, the cells were treated with PBS containing 0.5% bovine serum albumin and 0.15% glycine for 1 h and incubated with anti-activate Bak or Bax antibody overnight at 4°C. The cells were incubated with Alexa Fluor 488-conjugated secondary antibody for 1 h at room temperature, and examined under a Leica DC 300F fluorescence microscope.

### RNA interference

MCF-7 cells were seeded in six-well plates and transfected at 50% confluency with 100 nM of control non-specific siRNA or Bak- and Bax-targeted siRNAs using OligofectAMINE. Twenty-four hours after transfection, the cells were treated with DMSO (control) or specified concentrations of WA for 24 h. The cells were then collected and processed for immunoblotting and analysis of cytoplasmic histone-associated DNA fragmentation.
